# M10, a Myricetin-3-O-b-D-Lactose Sodium Salt, Prevents Ulcerative Colitis Through Inhibiting Necroptosis in Mice

**DOI:** 10.3389/fphar.2020.557312

**Published:** 2020-09-11

**Authors:** Xiao-Ling Zhou, Juan Yang, Xian-Jun Qu, Jian Meng, Rong-Rong Miao, Shu-Xiang Cui

**Affiliations:** ^1^ Beijing Key Laboratory of Environmental Toxicology, Department of Toxicology and Sanitary Chemistry, School of Public Health, Capital Medical University, Beijing, China; ^2^ Department of Pharmacology, School of Basic Medical Sciences, Capital Medical University, Beijing, China

**Keywords:** myricetin, derivative M10, ulcerative colitis, necroptosis, IL-6/NF-κB pathway, TNF-α pathway

## Abstract

**Background:**

M10 is a derivative of Myricetin by adding a hydrophilic glycosylation group. Our previous study revealed that M10 by oral administration prevented colitis-associated colonic cancer (CAC) through attenuating endoplasmic reticulum stress in mice. In current study, we evaluated the inhibitory effects of M10 on ulcerative colitis in mice model, the mechanism of M10 in preventing colitis was further investigated.

**Methods:**

Mice model of ulcerative colitis was induced by continuous oral dextran sodium sulfate (DSS). M10 was given gavage once a day for 12 consecutive weeks. Disease activity index (DAI) was recorded by analyzing the symptoms of colitis. Intestinal barrier was analyzed by the Immunoﬂuorescence staining assay. The structure of microvilli of intestinal epithelial cells was analyzed under Transmission electron microscopy (TEM). TEM assay was also performed to determine the formation of necroptosis in the colonic epithelium with ulcerative colitis. We performed Western blotting assay to analyze the IL-6 and NF-κB pathways, as well as the cytokine cascades related to TNF-α signaling pathway during necroptosis.

**Results:**

M10 by oral administration demonstrated a prevention of ulcerative colitis, showing a significant decrease of DAI as compared to the model mice. Pathological analysis indicated that M10 attenuated the degree of colonic inflammation in colonic tissues. M10 restored the structures of intestinal barrier damaged by DSS. M10 prevented the activation of the IL-6 and NF-κB signaling pathways in the inflamed colonic epithelium. Further, M10 prevented necroptosis in the inﬂamed colonic mucosal cells through down-regulating the TNF-α pathway. Importantly, M10 demonstrated higher activities in preventing ulcerative colitis than Myricetin and control drug Mesalazine.

**Conclusions:**

Myricetin derivative M10 prevents chronic ulcerative colitis through inhibiting necroptosis. M10 could be developed as a promising drug for the treatment of chronic ulcerative colitis.

## Introduction

Ulcerative colitis (UC) is characterized by chronic and relapsing inflammation in the mucosa of colorectum (Ungaro et al., 2017). It has emerged as a major public health concern due to high incidences particularly in Western countries. Globally, the incidence of UC is ~20 per 100,000 per year and the prevalence at ~250 cases per 100,000 persons (Muthas et al., 2017). Clinical symptoms of UC include diarrhea, bloody stools, abdominal pain and weight loss, etc (Podolsky and Xavier, 2007). The pathogenesis of UC has been considered to be related to the genetics, chronic infection, immunity disorder, environmental factors, and loss of intestinal mucosal barrier (Dutta and Chacko, 2016). Current therapeutic drugs such as aminosalicylates and Mesalazine are based on the mitigation of symptoms, including inflammatory remission and healing of intestinal mucosal manifestations. However, these drugs are uncertain, as the response to the treatment often diminishes over time, resulting in disease complications. A review paper which summaried clinical data from 1980 to 2018 indicates that Mesalazine is not superior to the placebo in relieving the symptoms of abdominal pain, and no significant advantage of reducing defecation frequency per day and immune cell infiltration and improving stool consistency as compared with the placebo (Zhang F. M. et al., 2019). In addition, as the aminosalicylates drugs, Mesalazine and sulfasalazine have many adverse effects such as the induction of dilated cardiomyopathy and severe heart failure (Sehgal et al., 2018). Although these adverse effects could be tolerable and manageable, they largely reduced the quality of life with long-time use. Therefore, better therapeutic drugs are still needed for the treatment of UC.

M10 is a derivative of myricetin-3-O-b-D-lactose sodium salt designed by adding a hydrophilic glycosylation group. Our previous studies indicated that M10 has the activity of prevnting colitis-associated carcinoma (CAC). M10 prevented CAC through reducing endoplasmic reticulum (ER) stress-induced autophagy in colonic mucosal cells (Wang et al., 2018). Chemically, Myricetin exhibits substantial limitations, such as poor water solubility (< 100 ng/ml) and low bioavailability (Yao Y. et al., 2014). After adding a hydrophilic glycosylation group, M10 exhibited fully water-solubility and high stability in Wistar rat plasma and liver microsomes (Zhu et al., 2019). We thus suggest that M10 demonstrate higher efficacy than its mother drug Myricetin in the prevention of chronic inflammation. Herein, we evaluated the efficacy of M10 in the DSS-induced UC mice, a reproducible and widely used UC model (Eichele and Kharbanda, 2017). M10 by oral administration demonstrated a strong inhibitory effect on UC through preventing the IL-6 and NF-κB pathways. The mechanism of M10 action might associate with its activity in the inhibition of necroptosis in the inflamed colonic epithelium through down-regulating the TNF-α signaling pathway. Given these biological properties, we suggest that M10 might meet the basic requirements as a therapeutic drug for the treatment of human chronic UC.

## Materials and Methods

### M10 and Other Chemicals

M10 (purity ≥ 98%) was provided by Dr. Li in Marine Biomedical Research Institute of Qingdao. M10 was fully dissolved in water. The design, synthesizing and analysing the properties of derivatives of Myricetin were published in the reference (Zhu et al., 2019). The following screening assays for M10 were carried out by Dr. Wang as shown in the reference (Wang et al., 2018). Myricetin (purity > 99%) was purchased from Sigma Chemical Co. (St. Louis, MO). Mesalazine was purchased from Anhui Dongsheng Pharmaceutical Co., Ltd. Myricetin and Mesalazine were prepared in 0.5% sodium carboxymethyl cellulose solvents (Solarbio, Beijing, China) before use.

### Mice UC Model and Drug Treatment

The animal protocol was approved by Capital Medical University Institutional Animal Care and Use Committee (AEEI-2016-076). Experiments were performed in accordance with the approved guidelines. Male C57BL/6 mice of 6-8 weeks age were purchased from Charles River Laboratories (Beijing, China). Mice were caged under controlled room temperature, humidity and light (12/12 h light/dark cycle) and allowed unrestricted access to standard mouse chow containing 52% carbohydrate, 12% fat, 23% protein, 4% fiber, 6% ash, and 3% moisture and tap water (Meidenbauer et al., 2014).

A total of 70 C57BL/6 mice were adapted to the facility for one week, and then randomly divided into following groups (n = 10 for each group): Group 1: normal mice (oral distill water); Group 2: chronic colitis model mice induced by DSS (oral distill water); Group 3: mice exposed to DSS treated with 100 mg/kg of Myricetin; Group 4–6: mice exposed to DSS treated with 25, 50, and 100 mg/kg of M10, respectively; Group 7: mice exposed to DSS treated with 100 mg/kg of Mesalazine. Mice model of UC was established as described previously (Wang et al., 2018). Mice were received water containing 1.0% DSS (36–50 kD, MP Biomedicals, LLC, Santa Ana, CA) for 7 days, and then given regular water for 14 days, followed by three additional DSS cycles. Mice received either vehicle or drugs by gavage six times per week for 12 consecutive weeks. Mice were checked and weighed daily for any signs of illness. The pathological process of UC was assessed by disease activity index (DAI) according to a standard scoring system (Cooper et al., 1993).

### Tissue Processing and Colorectal Length Measuring

After 4 DSS cycles, mice were sacrificed and whole colorectum was harvested. Taken photos and then calculated the length of colorectum by Image J software. Sections of colorectal tissues were snap-frozen in liquid nitrogen. Other sections were placed in 4% paraformaldehyde for histopathology analysis.

### Histopathologic Analysis

The sections of colorectal tissues were immediately dehydrated and then were embedded in paraffin. The paraffin intestinal samples were cut and stained with hematoxylin and eosin (H&E) for inﬂammation analysis. The degree of colorectal inﬂammation was graded according to the method in previous report (Dieleman et al., 1998).

### Transmission Electron Microscopy (TEM)

For TEM analysis, fresh colorectal tissues with 2 mm^3^-thick were transferred into 2.5% glutaraldehyde at room temperature for 2 h and then fixed overnight in refrigerator. The samples were rinsed several times with phosphate buffer and then fixed with 1% osmium tetroxide for 1 h. After rinsing with distilled water, the samples were dehydrated with different ethanol concentrations. After dehydration, a solution of propylene oxide and resin (1:1) was used for infiltration analysis. The samples were embedded in resin and then cut into ultra-thin sections (100 nm). Staining procedure was carried out by using 4% uranyl acetate (20 min) and 0.5% lead citrate (5 min) (Chen et al., 2018). TEM assay was performed under a transmission electron microscope (Hitachi, Tokyo, Japan).

### Western Blotting Assay

Equal amounts of total proteins from the supernatant of colorectal mucosa were extracted and then resolved by SDS-PAGE. The proteins were electro-transferred onto polyvinylidene ﬂuoride (PVDF) membranes. After blocking in 5% non-fat milk for 1 h at room temperature, membranes were immunoblotted overnight at 4°C with following primary antibodies: IL-6 (GB11117, Servicebio), JAK2 (3230, CST), p-JAK2 (3776, CST), STAT3 (10253, Proteintech), p-STAT3 (9145, CST), TNF-α (17590, Proteintech), caspase 8 (13423, Proteintech), RIPK1 (17519, Proteintech), p-RIPK1 (31122, CST), RIPK3 (17563, Proteintech), p-RIPK3 (ab195117, Abcam), MLKL (66675, Proteintech), p-MLKL (ab196436, Abcam), β-actin (A1978, Sigma). We determined the expressions and activations of kinases in the NF-κB pathway by analyzing the NF-κB Pathway Sampler Kit (9936T, CST). This kit contained the antibodies of NF-κB p65/RelA, phospho-NF-κB p65, IκBα, phospho-IκBα, IKKα, IKKβ, phospho-IKKα/β. PVDF membranes were washed in 0.1% Tween-20/Tris-buffered saline and then incubated with horseradish peroxidase-conjugated secondary antibodies included anti-mouse IgG (ZB-2305, ZSGB) and anti-rabbit IgG (ZB-2301, ZSGB). The bound antibodies were visualized using an enhanced chemiluminescence reagent (Millipore) and quantified by analyzing the densitometry using FluorChem FC3 image analyzer (Molecular Devices). Densitometric analyses of bands were normalized with β-actin functioning as a loading control.

### Cytokine Measurement by ELISA Assay

Colorectal mucosa (100 mg) were homogenized in 1 ml homogenization buffer (0.1 mol/l phosphate buffer, pH 7.4 containing 1 mmol/l EDTA and 10 μmol/l indomethacin) with polytron-type homogenizer. Homogenates were centrifuged at 14,000*g* for 10 min at 4°C. The supernatants were stored at −80°C. Levels of cytokines IL-4 (SEA077Mu), IL-10 (SEA056Mu), soluble TNF-α (SEB499Mu), IL-1 receptor antagonist (IL1RA) (SEA223Mu), and TGF-β (SEA124Mu) in were determined using luminex ELISA assay kits (Cloud-Clone Corp, China) according to manufacturer’s protocols.

### Immunoﬂuorescence Staining Analysis of the Intestinal Barrier

Colorectal tissues were fixed in paraformaldehyde, L-Lysine and NaIO4 (Periodate-Lysine-Paraformaldeyde buffer) and then were washed, dehydrated in 20% sucrose and included in optimal cutting temperature (OCT) cryo-embedding medium. Sections (10 µm-thick) were rehydrated with 0.1 M Tris HCl pH 7.4 buffer, blocked with 0.3% Triton X-100, Tris-HCl buffer 0.1 M containing 2% FBS. Slides were incubated with anti-ZO-1 FITC (sc-33725, Santa Cruz) for 2 h. Nuclei were counterstained with 4’,6-diamidino-2-phenylindole (DAPI) (C1002, Beyotime) and mounted with Vectashield (Burrello et al., 2019). Images were captured under a ﬂuorescence microscope.

### Statistical Analysis

Statistical analysis was conducted by using GraphPad Prism 7.0. All values are expressed as mean ± standard deviation (SD). Multiple group comparisons were analyzed by one-way analysis of variance (ANOVA) and multiple between-group comparisons were performed by using the LSD method. *p* < 0.05 was considered significant difference.

## Results

### M10 by Oral Administration Prevented DSS-Induced Chronic Ulcerative Colitis

**Figure 1:**
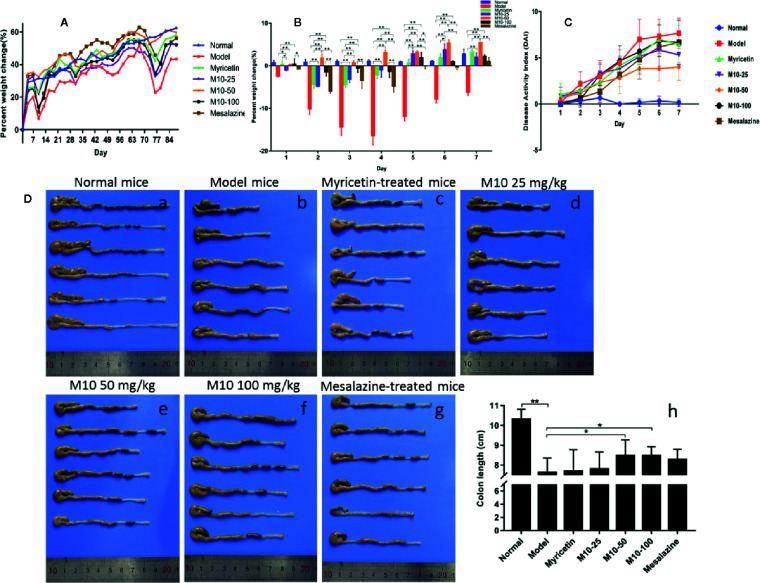
M10 ameliorated dextran sodium sulfate (DSS)-induced ulcerative colitis in mice. **(A)** Mice body changes during colitis induced by four DSS cycles exposure. **(B)** Mice body changes in various treatments during first 7 days regular water in fourth DSS cycle. As compared to normalized normal mice (blue color), model mice continued body weight loss for 3–4 days, and then slowly recovered, while body weight in the M10-treated mice did not significantly loss (50 mg/kg-treated mice) or recovered earlier after exposure to distilled water (25- and 100 mg/kg-treated mice). **(C)** Administration of M10 significantly reduced DAI scores during colitis in fourth DSS cycle. Normal mice did not demonstrate any symptoms of illnesses. Model mice were scored the highest DAI. **(D)** Length of colorectums in different mice groups. (a) Normal mice; (b) Model mice; (c) Myricetin-treated mice; (d-f) M10-treated mice by 25, 50, and 100 mg/kg, respectively; (g) Mesalazine-treated mice; (h) Statistical analysis of length of colorectums in different mice groups. Each dot represents six mice (n = 6). *p < 0.05, **p < 0.01 *vs.* model mice as well as Myricetin- or Mesalazine-treated mice.

Normal mice did not present any syndromes of diseases, and their weight gradually increased in a period of 12 week’s oral water. Model mice presented serious syndromes of UC, such as diarrhea, hair bristling, weight loss and bloody stools. During one week of DSS exposure, mice weight lost about 20%–30% relative to the day prior to DSS administration. The symptoms of UC were then gradually ameliorated during 2 weeks of regular water in the mice with ulcerative colites. Total of 4 DSS cycles were carried out to induce ulcerative colitis. During 2 week’s regular water, model mice continued to lose weight, and then their weight was slowly recovered. As compared to model mice, body weight in M10-treated mice recovered earlier 3–4 days after oral distill water. As shown in **Figure 1A**, the averages of weight calculated by increased percentage in all three doses of M10- and Myricetin-treated mice were around normal mice in the column plots. Mesalazine weakly prevented the DSS-induced weight loss. **Figure 1B** showed the body changes of mice in various treatments during first 7 days regular water in fourth DSS cycle. As compared to normalized normal mice (blue color), model mice continued body weight loss for 3-4 days, and then slowly recovered, while body weight in M10-treated mice did not significantly loss (50 mg/kg-treated mice) or recovered earlier after exposure to distilled water (25- and 100 mg/kg-treated mice). The severities of ulcerative colitis were scored by DAI. As compared to normal mice, all mice exposed to DSS were recorded various levels of DAI. Model mice were scored the highest DAI, Mesalazine- and Myricetin-treated mice exhibited moderate levels of DAI. M10-treated mice were scored the lowest level of DAI. In addition, M10-treated mice recovered sooner than other groups of mice during the 2 week’s regular water (**Figure 1C**). The average length of colorectums was 10.3 cm in normal control mice (**Figure 1D-a**). DSS-induced ulcerative colitis resulted in a shortening of colorectal length. As shown in **Figure 1D-b**, model mice scored the shortest length by 7.6 cm/colon with swelling colorectal tissues, while M10 by 25, 50, and 100 mg/kg prolonged the lengths of colorectums to 7.8, 8.5, and 8.5 cm/colorectum (**Figure 1D-d-f**), respectively (*p* < 0.05 *vs.* model mice). The average lengths of colorectums were 7.7 cm (**Figure 1D-c**) and 8.3 cm (**Figure 1D-g**), respectively, in Myricetin- and Mesalazine-treated mice (**Figure 1D-h**). Statistical analyses indicated significant differences between M10 and Myricetin (**Figure 1D-h**).

### Oral Administration of M10 Ameliorated the Severity of Chronic Inflammation in Colorectal Tissues of UC Mice Model

**Figure 2:**
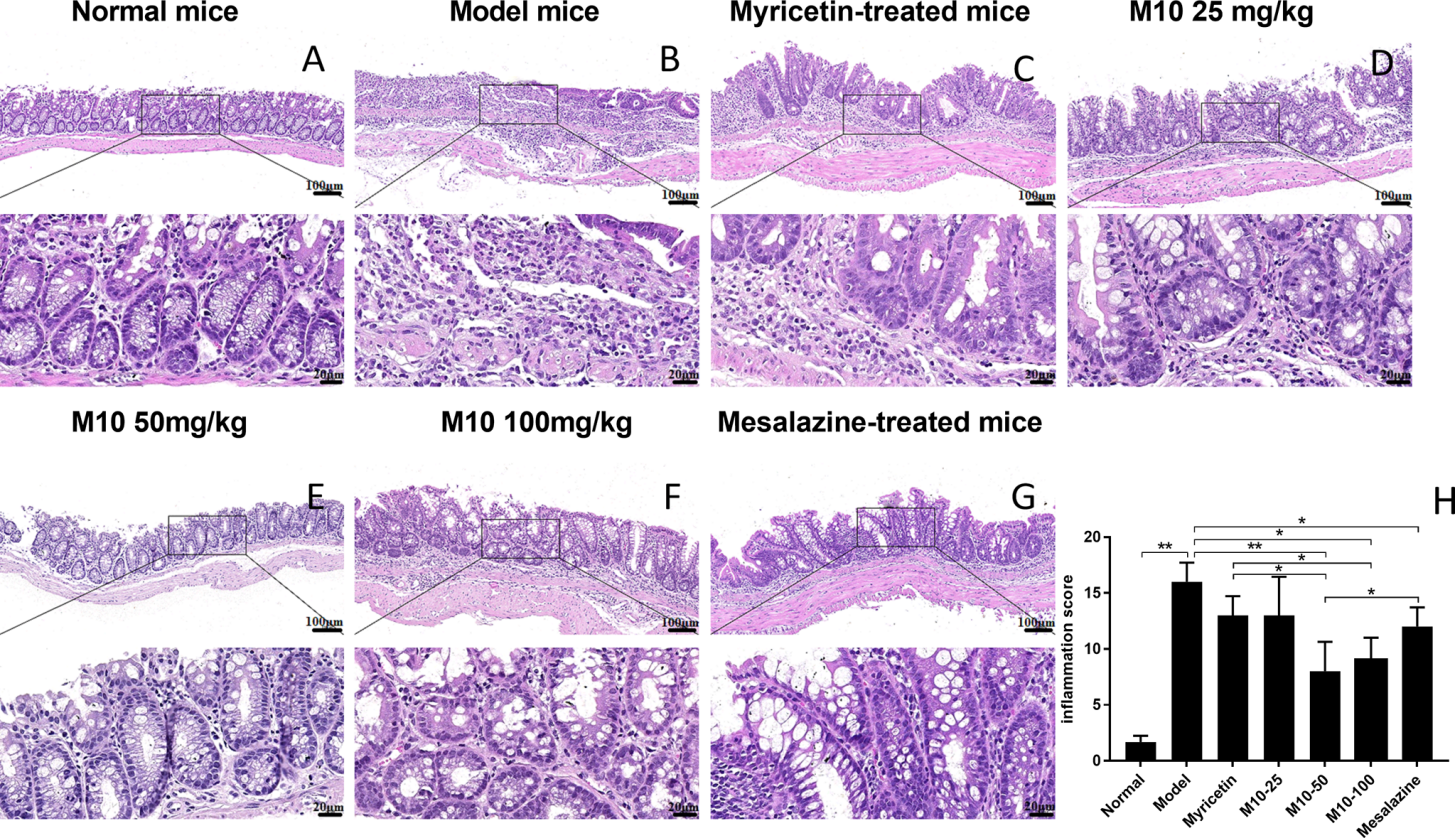
M10 decreased the severity of ulcerative colitis in colorectal tissues. The representative of H&E-stained colorectal tissues of normal mice **(A)**; Model mice **(B)**; Myricetin-treated mice **(C)**; M10-treated mice by 25, 50, and 100 mg/kg **(D–F)**, respectively; and Mesalazine-treated mice, ×100 (above), ×400 (bottom) **(G)**; The pathological scores of chronic inflammation in colorectal tissues in each group **(H)**, *p < 0.05, **p < 0.01 *vs.* model mice as well as Mesalazine-treated mice.

Histopathologic analysis showed that DSS-induced lesions in colorectum in 100% of model mice, but less severity in M10-treated mice (**Figure 2**). Observed inflammatory lesions included different degrees of structural changes ranging from swelling and degeneration of villous epithelial to extensive denudation and collapse of villi, surface erosion with exuberant inﬂammatory exudate, patchy reepithelization, lamina propria fibrosis with acute and chronic inflammatory immunocytes infiltration and submucosa edema (**Figure 2B**, model mice). M10 by oral administration strongly ameliorated the severity of chronic inflammation in colonic tissues (**Figures 2D–F**, *p* < 0.01 *vs.* model mice), and its efficacy in prevention of inflammation was significantly higher than Myricetin (**Figure 2C**, *p* < 0.05) and Mesalazine (**Figure 2G**, *p* < 0.05).

### Oral Administration M10 Prevented DSS-Induced Collapse of Intestinal Epithelial Barrier

**Figure 3:**
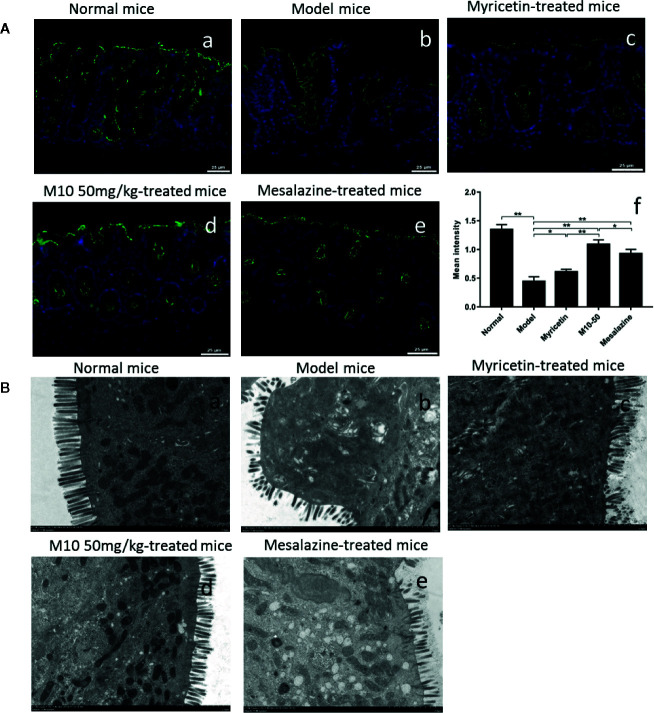
M10 prevented the collapse of intestinal epithelial barrier during colitis. **(A)** Intestinal epithelial barrier analysis by Immunoﬂuorescence staining assay. (a) Normal mice; (b) Model mice; (c) Myricetin-treated mice; (d) M10-treated mice by 50 mg/kg; (e) Mesalazine-treated mice; (f) Statistical analysis the average of fluorescence density in the intestinal epithelial barrier in each group, *p < 0.05, **p < 0.01 *vs.* model mice as well as Mesalazine-treated mice. **(B)** Transmission electron microscopy (TEM) analyzed the colonic epithelial villi in normal mice (a); Model mice (b); Myricetin-treated mice (c); M10-treated mice by 50 mg/kg (d); Mesalazine-treated mice (e). As compared to normal mice, model mice exhibited villous shedding and disordered arrangement. The villi of colonic epithelial cells in M10-treated mice arranged neatly without shedding.

Normally, intestinal barrier contributes to gut homeostasis through a well-structured epithelial layer, the secretion of antimicrobial peptides, and the formation of a multi-layer mucous barrier (Rescigno, 2011). The intestinal epithelial barrier mainly consists of villous epithelial and intercellular junctions. Tight junction (TJ) is the apical-most intercellular structure in villous epithelium, accounting for cell-cell adhesion, polarity, and the permeability barrier to paracellular transport of the solutes. We performed the immunoﬂuorescence staining assay to analyze intestinal barrier by determining the junction protein zonula occludens-1 (ZO-1). Normal mice demonstrated the intestinal epithelial barrier with regular architecture of tight junction protein ZO-1 (**Figure 3A-a**). Model mice were seen the broken barrier with discontinuous and weak stained ZO-1 (**Figure 3A-b**). M10 obviously prevented DSS-induced collapse of intestinal epithelial barrier, demonstrating the intestinal epithelial barrier, on the whole, continued stained ZO-1 architecture (**Figure 3A-d**). The records of average fluorescence density in intestinal epithelial barrier of M10-treated mice were significantly higher than model mice (*p* < 0.01*)*. Statistical analysis also showed the differences betwee Myricetin (**Figure 3A-c**, *p* < 0.01) and Mesalazine (**Figure 3A-e**, *p* < 0.05) (**Figure 3A-f**).

Transmission electron microscopy (TEM) analysis further confirmed the effect of M10 in the protection of intestinal barrier. In normal mice, the villi of colonic epithelial cells were completely and arranged neatly (**Figure 3B-a**). Model mice had different degrees of villous shedding and disordered arrangement (**Figure 3B-b**). In contrast, M10-treated mice demonstrated the villi arranged neatly without obvious shedding (**Figure 3B-d**). The activity of protecting the villi of intestinal barrier was higher than that of Myricetin (**Figure 3B-c**) and Mesalazine (**Figure 3B-e**).

### M10 Down-Regulated the IL-6 and NF-κB Pathways in the Inflamed Colonic Epithelium

**Figure 4:**
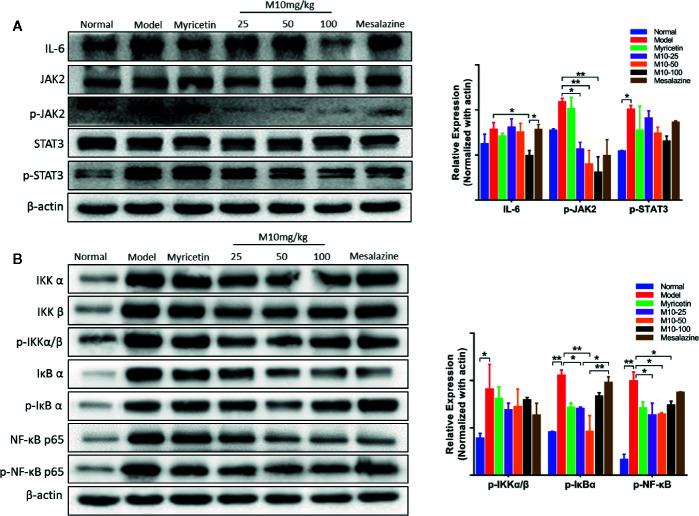
M10 down-regulated the IL-6 and NF-κB signaling pathways in the inflamed colonic epithelium. **(A)** M10 with 25, 50, and 100 mg/kg suppressed the IL-6 signaling pathway in colonic mucosal cells. **(B)** M10 down-regulated NF-κB signaling pathway. N = 6. *p < 0.05, **p < 0.01 *vs.* model mice as well as Myricetin- and Mesalazine-treated mice.

IL-6, a multi-effective cytokine, plays an important role in the pathogenesis of UC (Jones and Jenkins, 2018). A large number of studies revealed that blocking the processes of the IL-6 signal pathway could down-regulate multiple proinflammatory factors and consequently ameliorated the symptoms of chronic ulcerative colitis (Yao X. et al., 2014). Our results showed that M10 significantly reduced the expression levels of IL-6 (*p* < 0.05 *vs.* model mice), phosphorylated-JAK2 (*p* < 0.05 or 0.01 *vs.* model mice) and phosphorylated-STAT3, but not JAK2 and STAT3 in colonic epithelium (**Figure 4A**). In contrast, Myricetin and Mesalazine did not significantly regulate the IL-6 signal pathway.

It is well known that NF-κB plays a crucial role in promoting ulcerative colitis processes (Gambhir et al., 2015). M10 prevented chronic colitis through reducing the NF-κB signaling pathway. Western blotting analysis showed that the levels of phosphorylated-NF-κB in 25 mg/kg, 50 mg/kg and 100 mg/kg of M10 were inhibited to 63.6%, 64.6%, and 74.2%, respectively, of model mice. Myricetin and Mesalazine weakly decreased the level of phosphorylated-NF-κB to 71.2% and 87.9%, respectively, of model mice (**Figure 4B**). Furthermore, the levels of phosphorylated-IKKα/β (*p* < 0.05 *vs.* model mice) and phosphorylated-IκBα (*p* < 0.01 *vs.* model mice) were significantly suppressed by 13%–24% and 21%–56%, respectively, in the colonic epithelium.

**Figure 5:**
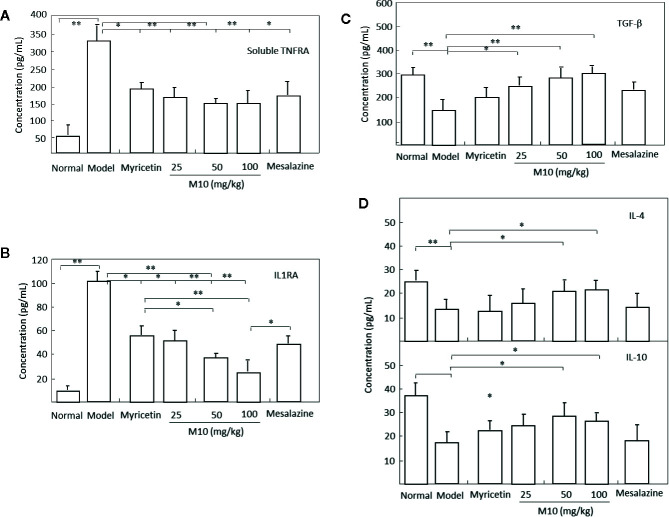
M10 reduced inflammatory cytokines and increased anti-inflammatory cytokines in the inflamed colonic epithelium. M10 with 25, 50, and 100 mg/kg resulted to the prevention of release of proinflammatory cytokines soluble TNFR1 **(A)** and IL1RA **(B)**, and a concomitant increase of anti-inflammatory cytokines TGF-β **(C)**, and IL-4 and IL-10 **(D)** in the inflamed colonic epithelium. N = 6. *p < 0.05, **p < 0.01 *vs.* model mice as well as Myricetin- and Mesalazine-treated mice.

It is noted that cytokines IL-10 and IL-4, TNF receptors, IL-1 receptor antagonists, and transforming growth factor (TGF)-β share a multitude of proinflammatory properties and appear to be critical to the amplification of mucosal inflammation (Abron et al., 2018; Tatiya-Aphiradee et al., 2018). M10 prevented the release of proinflammatory cytokines soluble TNFR1 and IL1RA (**Figures 5A, B**); and demonstrated a concomitant increase of anti-inflammatory cytokines IL-4, IL-10 and TGF-β in the inflamed colonic epithelium. Myricetin and Mesalazine exhibited weak effects in the regulation of these cytokines in the inflamed colonic epithelium (**Figures 5C, D**). These results indicate that M10 treatment results to a reduction in colorectal inflammatory cytokine levels and an increase in anti-inflammatory cytokines, probable due to the regulation of IL-6/NF-κB pathways in the inflamed colonic epithelium.

### M10 Prevented the Formation of Necroptosis in the Inflamed Colonic Epithelium

**Figure 6:**
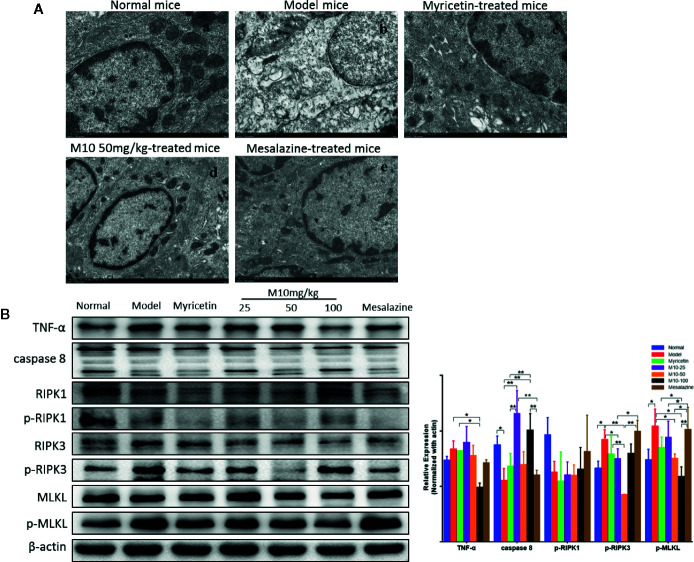
M10 prevented necroptosis during ulcerative colitis. **(A)** M10 prevented DSS-induced ultrastructural changes of colonic epithelial cells. (a) Normal mice; (b) Model mice; (c) Myricetin-treated mice; (d) M10-treated mice by 50 mg/kg; (e) Mesalazine-treated mice. **(B)** M10 reduced the expression of necroptosis-related proteins, TNF-α, caspase-8, RIPK1, p-RIPK1, RIPK3, p-RIPK3, MLKL, p-MLKL. N = 6. *p < 0.05, **p < 0.01 *vs.* model mice.

Necroptosis is a programmed necrotic cell death, defined as a distinct form of cell death that is caspase-independent through receptor-interacting serine/threonine kinase RIPK1/RIPK3 complex. Necroptosis is involved in many pathological processes of diseases, including chronic colitis (Royce et al., 2019). To determine the effect of M10 in preventing necroptosis in the inflamed colonic epithelium, we performed the transmission electron microscopy (TEM) assay to determine the necroptosis in the inflamed colonic epithelium by analyzing the ultrastructures of colonic epithelium (**Figure 6A**). Normal mice demonstrated totally normal nuclei, mitochondria, endoplasmic reticulum and Golgi body, and other organelles in the colonic epithelium (**Figure 6A-a**). Model mice displayed dilated nuclear membrane, mitochondria swelling, and the appearance of translucent cytosol, which were typical morphological characteristics of necroptosis (**Figure 6A-b**). M10 prevented DSS-induced necroptosis, showing apparently integrity and normal structures of nuclei, mitochondria, endoplasmic reticulum, Golgi body, and cell cytosol (6A-d). Compacted and segregated chromatins and membranes enclosed with cytoplasmic blebs were not seen as frequently as in the model mice. The inhibitory effect of M10 on DSS-induced necroptosis was more visible than that in Myricetin-treated mice (**Figure 6A-c**) and Mesalazine-treated mice (**Figure 6A-e**).

We further analyzed the consequent changes of signal pathway in M10-treated colonic epithelium. It is noted that activation of TNF-α signaling pathway plays a critical role during necroptosis and pathogenesis of chronic colitis. Upon TNF-α stimulation, RIPK1, FADD, and CYLD are recruited to TNFR1 to form a protein complex. Subsequent deubiquitylation and phosphorylation events lead to RIPK1 phosphorylation (de Almagro et al., 2017). When caspase-8, the upstream signaling of necroptosis, is absent or inhibited, the phosphorylated RIPK1 regulates the formation of a necrosome that consists of RIPK1, RIPK3 (Zhang C. et al., 2019). MLKL is then recruited to the RIPK1/RIPK3 complex and phosphorylated by p-RIPK3. Phosphorylated MLKL forms oligomers and translocates to intracellular membrane where it binds to phosphatidylinositol lipids, then leading to pores and finally disrupting cellular membrane integrity (Wang et al., 2014). In the model mice of ulcerative colitis, the expression of TNF-α was increased; conversely the level of caspase 8 was decreased in the inflamed colonic epithelium (*p* < 0.05 *vs.* normal mice). Consequently, phosphorylated-RIPK3 (*p* < 0.05 *vs.* normal mice) and phosphorylated-MLKL, the executor of necroptosis, were increased (*p* < 0.05 *vs.* normal mice), while M10-treated colonic epithelium demonstrated the prevention of TNF-α (*p* < 0.05 *vs.* model mice), phosphorylated-RIPK3 (*p* < 0.01 or 0.05 *vs.* model mice), and phosphorylated-MLKL (*p* < 0.05 *vs.* model mice). Myricetin and Mesalazine weakly inhibited the expressions of TNF-α, phosphorylated-RIPK3, and phosphorylated-MLKL. However, a significant difference of phosphorylated-RIPK1 was not seen among the above mice groups (**Figure 6B**, right). These results suggest that M10 might prevent the necroptosis in the inflamed colonic epithelium through down-regulating the TNF-α signaling pathway.

## Discussion

Currently, the pharmacotherapies for UC are mainly aminosalicylates sulfasalazine and Mesalazine, glucocorticoids and immunomodulators. However, the efficacies of these drugs are uncertain (Nielsen et al., 2016). In addition, long-term administration of sulfasalazine and Mesalazine could induce many side effects such as chronic hepatitis, liver fibrosis, renal complications, pulmonary toxicity, blood dyscrasias, sexual dysfunction, and even rarely pancreatitis (Durando et al., 2017; Sehgal et al., 2018). Monoclonal antibodies are associated with high risk of losing response after long term use (Moss et al., 2013). In our group, we designed M10 based on the structure of Myricetin by adding a hydrophilic glycosylation group for increasing water-solubility and the activity of anti-chronic inflammation. Our previous study revealed that M10 by oral administration prevented colorectal tumor through attenuating endoplasmic reticulum stress (Wang et al., 2018). However, the effect of M10 on UC has not been identified. We have noted that chronic colitis is the risk of colorectal tumor. Therefore, we further evaluated the activity of M10 in preventing chronic colitis. DSS model of colitis is widely used for investigating the pathogenesis and therapeutic response (Clapper et al., 2007). We thus employed colitis model induced by 4 DSS cycles. Our results showed that M10 by oral administration prevented weight loss, colitis symptom, and shortening of colonic length. Histopathologic analysis of colonic tissues showed the attenuated severity of chronic colitis. These results indicated that the design of M10 referenced Myricetin was successful. In addition, we compared the efficacy of M10 with Mesalazine, the most used drug for chronic colitis, in the prevention of chronic colitis. M10 demonstrated higher activity and lower DAI scores than Mesalazine in the prevention of ulcerative colitis.

UC has long been considered a complex pathogenesis associated to various factors, such as abnormal intestinal microbiota, dysregulated immune response, and a compromised intestinal epithelial barrier in genetically disposed of individuals. Recently, with the deepening of research on UC, people have gradually recognized the correlation between intestinal mucosal damage and the maintenance of UC (Zeissig et al., 2007). The rupture of epithelial tight junctions (TJs) barrier lead to the increase of permeability, followed by the permeation of pro-inflammatory molecules, and activated mucosal immune system, resulted to chronic inflammation and tissue damage. The precise regulation of intestinal barrier allows the maintenance of mucosal immune homeostasis and prevents the onset of uncontrolled chronic inflammation. ZO-1, a peripheral membrane protein forms narrow junction together with transmembrane proteins, is essential for maintenancing the intestinal mucosal barrier integrity (Turner, 2009). A recent study revealed that the expression of ZO-1 in UC patients was not only reduced as compared to that of healthy controls but also positively related to the intestinal mucosal healing (Tan et al., 2019). In this study, M10 by oral administration increased the expression of ZO-1, consequently resulting in the contribution to the restore of intestinal barrier.

Interleukin-6 (IL-6) is predominantly produced by macrophages and monocytes during acute inflammation and by T cells during chronic inflammation (Zheng et al., 2018). IL-6 signaling activates the tyrosine kinases JAK1, JAK2, and TYK2, which leads to the phosphorylation of signal transducers and activators of transcriptions 1 and 3 (STAT1 and STAT3) (Yao X. et al., 2014). Increasing evidence suggests that the signal STAT3 is an important intracellular signal transduction molecule because it is closely related to a number of inflammatory mediators and is normally present in the cytoplasm. It has been shown that STAT3 signaling is constitutively activated in the patients with UC and that the activation of STAT3 is responsible for triggering the innate immune response, leading to the production of proinflammatory mediators. The IL-6/STAT3 signaling regulates the survival of intestinal epithelial cells and plays an important role in the pathogenesis of UC (Saadatdoust et al., 2015). STAT family proteins, especially STAT3, are closely associated with nuclear transcription factor kappa B (NF-κB) signaling (Yu et al., 2009; He and Karin, 2011). We, therefore, explored whether M10 could alter the activation of the NF-κB pathway. Inactive NF-κB is located in the cytoplasm combined with an inhibitor of κBα (IκBα). Phosphorylation of IκBα by IκB kinase (IKK) complex in the presence of activating signals can induce the activation of NF-κB signaling (Napetschnig and Wu, 2013). Therefore, the activation of the NF-κB signaling plays crucial roles during chronic colitis (Neurath et al., 1996). M10 prevented both IL-6 and NF-κB pathways in the M10-treated colonic epithelium.

During colitis, cytokines serve as significant signals in the augmentation and perpetuation of inflammation. They are directly responsible for mucosal injury and tissue damage, and some consequently trigger disease-specific inflammatory responses in colitis (Tatiya-Aphiradee et al., 2018). In this study, M10 treatment prevented the release of proinflammatory cytokines soluble TNFR1 and IL1RA; and demonstrated a concomitant increase of anti-inflammatory cytokines IL-4, IL-10 and TGF-β in the inflamed colonic epithelium. We thus suggest that M10 treatment result to the prevention of the pathogenesis of chronic colitis, probable due to the regulatory effects of IL-6/NF-κB signaling pathways in the inflamed colonic epithelium.

Necroptosis has been considered to play critical roles during chronic colitis (Vandenabeele et al., 2010). Necroptosis was first detected in chronic inflammation induced by TNF-α (Degterev et al., 2005). In the TNF-α signaling pathway, TNF-α binds to and activates TNFR1. The activated TNFR1 then recruits TNF receptor-associated death domain (TRADD), receptor-interacting protein kinase-1 (RIPK1) and several ubiquitin E3 ligases to form complex I in which RIPK1 is polyubiquitinated and subsequent deubiquitinated to form complex II (Christofferson et al., 2014). When caspase-8 activity is suppressed, RIPK1 could promote necroptosis by interacting with receptor-interacting protein kinase-3 (RIPK3), which regulates the phosphorylation of mixed lineage kinase domain-like protein (MLKL). The phosphorylated MLKL monomers aggregate to form the oligomers and translocate to plasma membrane to execute necroptosis (Wang et al., 2014). This translocation of MLKL results to membrane perforation and subsequently releases damage-associated molecular patterns into extracellular environment and triggers necroptosis (Ito et al., 2016; Royce et al., 2019). In colitis model, we determined an inhibition of RIPK3 and MLKL during necroptosis in the inflamed epithelium, whereas a down-regulation of RIPK3 and MLKL was observed in the M10-treated epithelium. These results suggest that M10 might prevent necroptosis of epithelium during colitis through down regulation of TNF-α signaling pathway. Taken together, Myricetin derivative M10 prevents DSS-induced chronic colitis through inhibiting the formation of necroptosis by down-regulating the TNF-α signaling pathway. Long term use of M10 did not induce side effects in mice. We thus suggest that M10 could be developed as a promising drug for the treatment of chronic ulcerative colitis.

## Data Availability Statement

The raw data supporting the conclusions of this article will be made available by the authors, without undue reservation, to any qualified researcher.

## Ethics Statement

The animal study was reviewed and approved by Capital Medical University Institutional Animal Care and Use Committee.

## Author Contributions

X-LZ and JY performed all experiments related to animals. R-RM and JM provided the technical support and performed the statistical analysis. X-JQ wrote the manuscript. S-XC received the project and corrected the manuscript. All authors contributed to the article and approved the submitted version.

## Funding

This work was supported by the Natural Science Foundation of China (91629303/81673449/81872884/81973350) and the Beijing Natural Science Foundation Program and Scientific Research Key Program of Beijing Municipal Commission of Education (KZ201710025020/KZ201810025033).

## Conflict of Interest

The authors declare that the research was conducted in the absence of any commercial or financial relationships that could be construed as a potential conflict of interest.
